# Prediction of subcellular location of apoptosis proteins by incorporating PsePSSM and DCCA coefficient based on LFDA dimensionality reduction

**DOI:** 10.1186/s12864-018-4849-9

**Published:** 2018-06-19

**Authors:** Bin Yu, Shan Li, Wenying Qiu, Minghui Wang, Junwei Du, Yusen Zhang, Xing Chen

**Affiliations:** 1 College of Mathematics and Physics, Qingdao University of Science and Technology, Qingdao, 266061 China; 2 Artificial Intelligence and Biomedical Big Data Research Center, Qingdao University of Science and Technology, Qingdao, 266061 China; 3 School of Life Sciences, University of Science and Technology of China, Hefei, 230027 China; 4 College of Information Science and Technology, Qingdao University of Science and Technology, Qingdao, 266061 China; 5 School of Mathematics and Statistics, Shandong University at Weihai, Weihai, 264209 China; 6 School of Information and Control Engineering, China University of Mining and Technology, Xuzhou, 21116 China

**Keywords:** Apoptosis proteins, Subcellular localization, Pseudo-position specific scoring matrix, Detrended cross-correlation analysis coefficient, Local fisher discriminant analysis, Support vector machine

## Abstract

**Background:**

Apoptosis is associated with some human diseases, including cancer, autoimmune disease, neurodegenerative disease and ischemic damage, etc. Apoptosis proteins subcellular localization information is very important for understanding the mechanism of programmed cell death and the development of drugs. Therefore, the prediction of subcellular localization of apoptosis protein is still a challenging task.

**Results:**

In this paper, we propose a novel method for predicting apoptosis protein subcellular localization, called PsePSSM-DCCA-LFDA. Firstly, the protein sequences are extracted by combining pseudo-position specific scoring matrix (PsePSSM) and detrended cross-correlation analysis coefficient (DCCA coefficient), then the extracted feature information is reduced dimensionality by LFDA (local Fisher discriminant analysis). Finally, the optimal feature vectors are input to the SVM classifier to predict subcellular location of the apoptosis proteins. The overall prediction accuracy of 99.7, 99.6 and 100% are achieved respectively on the three benchmark datasets by the most rigorous jackknife test, which is better than other state-of-the-art methods.

**Conclusion:**

The experimental results indicate that our method can significantly improve the prediction accuracy of subcellular localization of apoptosis proteins, which is quite high to be able to become a promising tool for further proteomics studies. The source code and all datasets are available at https://github.com/QUST-BSBRC/PsePSSM-DCCA-LFDA/.

## Background

Protein maintains a highly ordered operation of the protection of the cell system [1]. At the cellular level, proteins work only in specific locations. It is necessary to fulfill the protein’s function that subcellular locations provide a specific chemical environment and set of interaction partners [2]. Apoptosis is cell physiological death which is closely related to intracellular control [3]. Cancer and autoimmune disease occurs when blocking apoptotic protein appears, ischemic damage or neurodegenerative disease occurs when unwanted apoptosis appears [4]. Studying proteins involved in the apoptotic process can help us understand the pathogenesis of the disease and provide a variety of therapeutic targets. It is very valuable to get information on apoptosis protein subcellular localization, which can help us understand the apoptosis proteins function, cell apoptosis mechanisms and drug development [5]. Therefore, it is a challenging task using the machine learning method to construct the protein subcellular location prediction model.

For nearly two decades, the research of protein subcellular localization prediction has been a hotspot in bioinformatics research. Currently, this topic has achieved some successes [6]. As for protein subcellular localization prediction methods, we found that the research focuses on the following two aspects: (1) Feature extraction of protein sequences. The main methods include N-terminal information prediction method [7, 8], protein amino acid composition prediction method [9, 10], pseudo-amino acid composition [11, 12], dipeptide composition [13, 14], grouped weight coding method [15], discrete wavelet transform [16, 17], position specific scoring matrix [18] and protein GO annotation [19, 20] and so on. (2) The choice of classification algorithm. At present, the commonly used prediction algorithms are increment of diversity [21], deep learning [22], K-nearest neighbor [23, 24], neural network [25], hidden Markov model [26, 27], Bayesian classifier [28, 29], support vector machine [30, 31] and ensemble learning [32–35]. Among them, SVM has fast computing speed, excellent ability to extract information, good generalization performance advantages, making the SVM become the first classifier choice for researchers.

Currently, apoptosis protein subcellular localization prediction has made great advancement. Zhou and Doctor [36] constructed 98 protein apoptosis protein dataset, using the amino acid composition and covariance discriminant method, the overall prediction accuracy reached 72.5% by jackknife test. Huang et al. [37] obtained the accuracy rate of 77.6% by combining the protein instability index with the support vector machine. However, the prediction capacity of this method was unbalanced. Especially, for other class proteins (exclude cytoplasmic, membrane and mitochondrial proteins), the prediction accuracy did not exceed 50%. Bulashevska and Eils [38] used Bayesian classifier based on Markov chain model to construct ensemble classifier, and the prediction accuracy of 98 apoptosis proteins was further improved by jackknife test. Zhang et al. [15] constructed a new apoptosis protein dataset of 225 proteins. They used encoding approach with grouped weight as feature extraction method for protein sequences and support vector machine as classifier (named as EBGW_SVM). The overall prediction accuracy was 83.1% using jackknife test. The feature extraction method of the protein sequence takes into account the distribution of residues with the same unique characteristic, but ignores the physical and chemical properties of the protein sequence. Chen and Li [39] constructed a dataset containing 317 apoptosis protein sequences and obtained higher prediction accuracy, which combined support vector machine and increment of diversity (named as ID_SVM) by using jackknife test. Similarly, Ding et al. [40] used the Fuzzy K-nearest neighbor (FKNN) algorithm and the overall prediction accuracy was 90.9% using CL317 dataset. Qiu et al. [41] used the DWT_SVM method to obtain high prediction accuracy rates of 97.5, 87.6 and 88.8% for CL317, ZW225 and ZD98 datasets, respectively by jackknife test. The above methods ignore the biological information of the protein sequence, so the prediction method of homologous similarity based on the protein sequence and protein functional domain is proposed. Yu et al. [42] proposed a novel pseudo-amino acid model which extracted the sequence characteristics of proteins using amino acid substitution matrices and auto covariance transformation and used support vector machine as classifier. The results of prediction accuracy obtained by jackknife test were 90.0 and 87.1% on the CL317 and ZW225 datasets, respectively. Liu et al. [43] used tri-gram encoding based PSSM as feature extraction method, then used SVM-RFE algorithm to reduce feature vectors, finally the best feature vectors were input to the SVM classifier. The prediction accuracy were 95.9, 97.8 and 96.9% on the CL317, ZW225 and ZD98 datasets, respectively. Dai et al. [44] treated the difference between the N-segment and C-segment of the protein in subcellular location prediction, and proposed a model based on golden ratio segmentation to improve subcellular localization prediction, and achieved a better predictive effect. Xiang et al. [45] introduced evolutionary-conservative information to represent protein sequences. Meanwhile, according to the proportion of golden section in mathematics, the position-specific scoring matrix (PSSM) is divided into several blocks. The overall accuracy of ZD98 and CL317 datasets were 98.98 and 91.11%, respectively by using SVM classifier. Liang et al. [46] combined the Geary autocorrelation function and detrended cross-correlation coefficient methods based on PSSM to extract the protein sequences from the CL317, ZW225 and ZD98 datasets. Under the jackknife test, the overall prediction accuracy were 89.0 84.4 and 91.8%, respectively.

Using only a feature is difficult to have a big breakthrough in the prediction of subcellular localization. At present, researchers usually combined multiple feature extraction methods of protein sequences to obtain more comprehensive protein sequence information. However, the feature vectors of the protein sequences obtained by fusing a variety of features are usually very high. High-dimensional data contains a lot of redundant information, which may seriously affect the performance of the classifier. Dimensionality reduction methods can help us eliminate redundant information and are widely used in data classification and pattern recognition. At present, many researchers introduce a variety of methods to reduce dimension in the subcellular localization prediction, such as SVD (singular value decomposition) [47], Backward feature selection [48], CFS (correlation-based feature selection) [49], Forward selection [50], PSO (particle swarm optimization) [51], mLASSO (multi-label least absolute shrinkage and selection operator) [52], GA (Genetic algorithm) [53] and so on.

In this paper, we presents a new method for predicting subcellular localization of apoptosis proteins, called PsePSSM-DCCA-LFDA. Firstly, obtain sequence information from apoptotsis protein sequences by combining PsePSSM algorithm and DCCA coefficient. Then, the LFDA method is used to reduce the dimension and noise information in the original high-dimensional space. Finally, using SVM as classifier to predict protein subcellular localization. By jackknife test, the optimal parameters of the model are determined under different ξ values, *S* values, different dimensionality reduction methods and selection of different dimensions, and established PsePSSM-DCCA-LFDA prediction model. Using the most rigorous jackknife test, the overall prediction accuracy are 99.7, 99.6 and 100%, respectively for CL317 dataset, ZW225 dataset and ZD98 dataset. The results show that the PsePSSM-DCCA-LFDA method can get better prediction effect than other existing methods.

## Methods

### Datasets

In this study, we use CL317 dataset and ZW225 dataset as the training datasets, which select optimal parameters of the prediction model. In addition, the ZD98 dataset is selected as an independent testing dataset that used to test the applicability of the prediction model. The CL317 dataset was constructed by Chen and Li [39], which contained 317 proteins classified into six compartments as cytoplasm proteins, mitochondrion proteins, nucleus proteins, membrane proteins, secreted proteins and endoplasmic reticulum proteins, each class containing 112, 34, 52, 55, 17, and 47 proteins, respectively. For CL317 dataset, distribution of sequence identity percentage are 40.1, 15.5, 18.9 and 25.6% with ≤40%, 41%-80%, 81%-90% and ≥91% sequence identity, respectively. The ZW225 dataset was constructed by Zhang et al. [15], which contained 225 proteins classified into four compartments as nuclear proteins, cytoplasmic proteins, mitochondrial proteins and membrane proteins, each class containing 41, 70, 25 and 89 proteins, respectively. For ZW225 dataset, distribution of sequence identity percentage are 52.9, 16, 16 and 15.1% with ≤40%, 41%-80%, 81%-90% and ≥91% sequence identity, respectively. The ZD98 dataset was constructed by Zhou and Doctor [36], which contained 98 proteins classified into four compartments as cytoplasmic proteins, plasma membrane-bound proteins, mitochondrial proteins and other proteins, each class containing 43, 30, 13 and 12 proteins, respectively. For ZD98 dataset, distribution of sequence identity percentage are 34.7, 30.6, 17.4 and 17.4% with ≤40%, 41%-80%, 81%-90% and ≥91% sequence identity, respectively. The protein sequences are extracted from SWISS-PROT database (version 49.5) in the three datasets, and we can find the accession numbers in the literatures [15, 36, 39].

### Pseudo-position specific scoring matrix (PsePSSM)

In order to obtain the evolutionary information of the protein sequences, the protein sequences of the CL317, ZW225 and ZD98 datasets are aligned with the non-redundant (NR) database (ftp://ftp.ncbi.nih.gov/blast/db/) using the PSI-BLAST program [54], and obtain the position specific scoring matrix (PSSM) [55] of the corresponding protein sequences. The NR database contains 85,107,862 protein sequences. We use three iterations and E-value is 0.001 in PSI-BLAST program. The BLOSUM62 matrix is used as substitution matrix for generating the PSSM. PSSM can be expressed for a protein sequence *P* as the following Eq. (1).

1 $$ {P}_{PSSM}=\left(\begin{array}{l}{E}_{1,1}\kern0.7em {E}_{1,2}\kern0.5em \cdots \kern0.6em {E}_{1,20}\\ {}\kern0.5em \vdots \kern1.5em \vdots \kern1.6em \vdots \kern1.6em \vdots \\ {}{E}_{i,1}\kern0.7em {E}_{i,2}\kern0.5em \cdots \kern0.6em {E}_{i,20}\\ {}\kern0.4em \vdots \kern1.5em \vdots \kern1.6em \vdots \kern1.6em \vdots \\ {}{E}_{L,1,}\kern0.7em {E}_{L,2}\kern0.5em \cdots \kern0.6em {E}_{L,20}\end{array}\right) $$

where *L* is total number of amino acids in the protein sequence, *E*_*i*, *j*_ represents the evolution information of amino acids in protein sequences. The rows of PSSM represent the corresponding amino acids positions in protein sequences, and columns of PSSM indicate the 20 amino acid types that may be mutated. The PSSM value ranges from − 9 to 11.

Since the length of the protein sequence in the CL317, ZW225 and ZD98 datasets is inconsistent, the corresponding PSSM dimension for the protein sequence in the dataset is different, which is difficult for our subsequent study. In this paper, PsePSSM [56] algorithm is used to extract the features of protein sequences, and the PSSM of different protein sequences is transformed into a uniform vector.

First, the elements of PSSM are normalized by Eq. (2), whose PSSM value ranges from 0 to 1.

2 $$ f(x)=1/\left(1+{e}^{-x}\right) $$

where *x* is the original PSSM value.

Then, a protein sequence can be expressed using PsePSSM as follows:

3 $$ {P}_{PsePSSM}={\left(\overline{P_1},\overline{P_2},\cdots, \overline{P_{20}},{\theta}_1^1,{\theta}_2^1,\cdots, {\theta}_{20}^1,\cdots, {\theta}_1^{\xi },{\theta}_2^{\xi },\cdots, {\theta}_{20}^{\xi}\right)}^T $$

where $\overline{P_j}=\sum \limits_{i=1}^L{P}_{i,j}/L\kern1.2em \left(j=1,2,\cdots, 20\right)$, ${\overline{P}}_j$represents the average score of the all amino acid residues which are mutated to *j* amino acid type in the protein *P*. ${\theta}_j^{\xi }=\frac{1}{L-\xi}\sum \limits_{i=1}^{L-\xi }{\left({P}_{i,j}-{P}_{\left(i+\xi \right),j\Big)}\right)}^2\kern1.5em \left(j=1,2,\cdots 20;\kern0.3em \xi <L,\kern0.3em \xi \ne 0\right)$, ${\theta}_j^{\xi }$ is order information of protein sequences, *j* is amino acid type, ξ is contiguous distance.

From the above, a protein sequence generates 20+20×ξ dimension feature vector using PsePSSM algorithm.

### Detrended cross-correlation analysis coefficient

According to the evolutionary information expressed by the protein sequence, we can obtain the corresponding position score-specific matrix (PSSM), as shown in eq. (1). In order to extract more protein sequence information from the PSSM matrix, the protein sequence information is extracted from the PSSM using the detrended cross-correlation analysis coefficient (DCCA coefficient) method [57–59]. DCCA coefficient is a method based on the trend covariance method, and the least squares linear fitting and trend elimination are carried out for nonstationary signals. The evolutionary information expressed in the form of PSSM is used as the attribute, and each amino acid is considered as one property. PSSM is considered to be the time series of all attributes. Since the size of the PSSM matrix for each protein sequence is *L*×20, we calculate the 20 columns in the PSSM matrix as 20 non-stationary time series [46, 60].

After normalizing the PSSM matrix using the eq. (2), for any two different columns {*m*_*i*_} and {*n*_*1*_} of PSSM (*i*=1,2,···,*L*), *L* is the length of protein sequence. First we use the Eq. (4) to calculate the new time series *M*_*k*_ and *N*_*k*_.

4 $$ \left\{\begin{array}{l}{M}_k=\sum \limits_{i=1}^k{m}_i\kern2.1em k=1,2,\cdots, L\\ {}{N}_k=\sum \limits_{i=1}^k{n}_i\kern2.3em k=1,2,\cdots, L\end{array}\right. $$

Then the time series *M*_*k*_ and *N*_*k*_ are divided into *L*-*S* segments which can be overlapped, each segment contains *S*+1 data, and then the least squares linearly fitting for each segment of the data to obtain the fitting values ${\tilde{M}}_{i,k}$ and ${\tilde{N}}_{i,k}$. Use the Eq. (5) to calculate the covariance of each segment.

5 $$ {f}_{xy}^2\left(S,i\right)=\frac{1}{S+1}\sum \limits_{k=i}^{i+S}\left({M}_k-{\tilde{M}}_{i,k}\right)\left({N}_k-{\tilde{N}}_{i,k}\right) $$

In particular, there are ${f}_{xx}^2\left(S,i\right)=\frac{1}{S+1}\sum \limits_{k=i}^{i+S}{\left({M}_k-{\tilde{M}}_{i,k}\right)}^2$,${f}_{yy}^2\left(S,i\right)=\frac{1}{S+1}\sum \limits_{k=i}^{i+S}{\left({N}_k-{\tilde{N}}_{i,k}\right)}^2$.

Next, the covariance of the *L*-*S* segments (whole time series) calculated by using the Eq. (6) is:

6 $$ {f}_{xy}^2(S)=\frac{1}{L-S}\sum \limits_{i=1}^{L-S}{f}_{xy}^2\left(S,i\right) $$

In particular, there are${f}_{xx}^2(S)=\frac{1}{L-S}\sum \limits_{i=1}^{L-S}{f}_{xx}^2\left(S,i\right)$,${f}_{yy}^2(S)=\frac{1}{L-S}\sum \limits_{i=1}^{L-S}{f}_{yy}^2\left(S,i\right)$.

Finally, the DCCA coefficients of two different time series {*m*_*i*_} and {*n*_*1*_} are calculated using Eq. (7).

7 $$ {\rho}_{DCCA}=\frac{f_{xy}^2(S)}{f_{xx}(S){f}_{yy}(S)} $$

As can be seen from Eq. (7), *ρ*_*DCCA*_ depends on the length *L* of the protein sequence and the length *S*+1 of the overlapping portion of each segment. Its value ranges from -1≤ *ρ*_*DCCA*_ ≤1, where 1 represents perfect cross-correlation, 0 indicates no cross-correlation, and − 1 represents perfect anti-cross-correlation [61]. Finally, the DCCA coefficient algorithm will generate a 190-dimensional feature vector for a protein sequence.

### Local fisher discriminant analysis

This paper uses a supervised dimensionality reduction method, local Fisher discriminant analysis (LFDA) [62]. LFDA has the form of embedded transformation, and it can be easily calculated by solving the generalized eigenvalue problem. Let the protein data matrix be *X* = [*x*_1_, *x*_2_, ⋯*x*_*n*_],*x*_*i*_ ∈ *R*^*d*^, where *n* is the number of samples of the protein, *d* is the dimension of the protein sequence feature extraction. *y*_*i*_ ∈ {1, 2⋯, *c*}, *n*_ℓ_ is the number of samples of the categoryℓ, $\sum \limits_{\mathrm{\ell}=1}^c{n}_{\mathrm{\ell}}=n$. The local within-class scatter matrix *S*^(*w*)^and the local between-class scatter matrix *S*^(*b*)^are calculated using Eqs. (8) and (9).

8 $$ {S}^{(w)}=\frac{1}{2}\sum \limits_{i,j=1}^n{W}_{i,j}^{(w)}\left({x}_i-{x}_j\right){\left({x}_i-{x}_j\right)}^T $$

9 $$ {S}^{(b)}=\frac{1}{2}\sum \limits_{i,j=1}^n{W}_{i,j}^{(b)}\left({x}_i-{x}_j\right){\left({x}_i-{x}_j\right)}^T $$

where

$$ {W}_{i,j}^{(w)}=\left\{\begin{array}{l}{A}_{i,j}/{n}_{\mathrm{\ell}}\kern0.9000001em if\kern0.4em {y}_i={y}_j=\mathrm{\ell}\\ {}0\kern3.499999em if\kern0.6em {y}_i\ne {y}_j\kern0.4em \end{array}\right. $$

$$ {W}_{i,j}^{(b)}=\left\{\begin{array}{l}{A}_{i,j}\left(\left(1/n\right)-\left(1/{n}_{\mathrm{\ell}}\kern0.1em \right)\kern0.1em \right)\kern0.7em if\kern0.4em {y}_i={y}_j=\mathrm{\ell}\\ {}1/n\kern3.499999em if\kern0.6em {y}_i\ne {y}_j\kern0.4em \end{array}\right. $$

It is worth noting that *A* is an affinity matrix, *A*_*i*, *j*_ ∈ *A* is the affinity between*x*_*i*_ and *x*_*j*_. In this paper, we use the affinity matrix*A*_*i*, *j*_ = exp(−‖*x*_*i*_ − *x*_*j*_‖/*σ*_*i*_*σ*_*j*_) defined by Zelnik-Manor and Perona [63]. ${\sigma}_i=\left\Vert {x}_i-{x}_i^{(K)}\right\Vert$ represents the local scaling of the surrounding *x*_*i*_ data samples, where ${x}_i^{(K)}$ is the *K* nearest neighbor of *x*_*i*_. The literature [63] proved that in the experiment for high-dimensional data, when *K* = 7, better results can be obtained, so this article selected *K* = 7.

Solve LFDA transformation matrix*T*_*LFDA*_

10 $$ {T}_{LFDA}=\underset{T\in {R}^{d\times r}}{\arg \max}\kern0.4em \left[ tr\left({\left({T}^T{S}^{(w)}T\right)}^{-1}{T}^T{S}^{(b)}T\right)\right] $$

Matrix after the dimension reduction becomes:

11 $$ Z={T}_{LFDA}^{\hbox{'}}X $$

Therefore, through the Eq. (11), we eliminate the redundant information contained in the high-dimensional data obtained after the original protein sequence feature extraction. In other words, the fusion PsePSSM algorithm and DCCA coefficient algorithm on the apoptosis protein sequence after the feature extraction matrix *X*, through the transformation matrix *T*_*LFDA*_, matrix *Z* is obtained after dimensionality reduction.

### Support vector machine

Support vector machine (SVM) is a supervised machine learning method based on statistical learning theory, which is proposed by Vapnik et al. [64]. Because of its excellent learning and generalization ability, especially the ability to deal with high dimensional sparse vector, it has become a hotspot in the field of data mining and machine learning. In recent years, SVM has also been widely used in the field of bioinformatics. In the field of proteomics research, it has been widely used to predict membrane protein types [65, 66], G protein-coupled receptors [67, 68], protein structure [69–73], protein-protein interaction [74–76], protein subcellular localization [77–80], protein post-translational modification sites [81–84] and other protein structure and function of the study.

SVM is used to solve a two-class classification problem. Set*D* = {(*x*_*i*_, *y*_*i*_)| *i* = 1, 2, ⋯, *n*}  is a training set, where*x*_*i*_ ∈ *R*^*d*^represent sample *i*, which has *d* dimension feature vectors, *y*_*i*_ ∈ {+1, −1}is class labels of sample *i*. SVM transforms a linearly indivisible sample of low-dimensional input space into high-dimensional feature space to make it linearly separable.

In this study, we choose the radial basis function (RBF) to perform prediction. Because RBF kernel function is the most widely used kernel function and its superiority for solving nonlinear problem [17, 18, 41–46], which is defined as follows:

12 $$ K\left({x}_i,{y}_j\right)=\exp \left(-\gamma {\left\Vert {x}_i-{y}_j\right\Vert}^2\right) $$

where *γ* is the kernel width parameter, *x*_*i*_ and *y*_*j*_ are the feature vectors of the *i*-th and *j*-th protein sequences, respectively. The egularization parameter *C* and the kernel parameter *γ* are optimized based on CL317 and ZW225 datasets by *K*-fold cross validation using a grid search strategy to obtain the highest overall prediction accuracy by using the LIBSVM software [85], which can be freely downloaded from http://www.csie.ntu.edu.tw/~cjlin/libsvm/. In this paper, *C* is allowed to take a value only between 2^−5^ and 2^15^, and *γ* only between 2^‐15^and 2^5^.

SVM is originally designed for two-class classification, but CL317, ZW225 and ZD98 are multi-class classification data. At present, three kinds of strategies can be solved multi-classification: one-versus-one (OVO), one-versus-rest (OVR) [86] and direct acyclic graph SVM (DAGSVM) [87]. LIBSVM software implements the “one-versu-one” (OVO) strategy for multi-class classification. The OVO strategy sets up a classifier between any two categories,so if *k* is the number of classes, then *k*(*k* − 1)/2 classifiers are constructed. During the testing phase, the test samples are submitted to all classifiers, *k*(*k* − 1)/2 classification results are obtained, and the final result is generated by voting. That is to say, the most voting category is the final class. It is worth noting that when there are two categories of voting the same results, we choose the class appearing first of the vote as the final category for the sake of simple operation.

### Performance evaluation and model building

In statistical prediction, there are four validation tests: self-consistency test, independent dataset test, k-fold cross-validation and jackknife test, which are often used to evaluate the prediction performance [78, 80]. In this paper, the jackknife test [88, 89] is used to examine the performance of the prediction model. The jackknife test requires testing each sample in the dataset. Specifically, each time one sample is selected as an independent test sample in the dataset, and the remaining samples are used as a training set to establish a prediction model until all the samples have been tested in the dataset.

We use four standard performance measures to evaluate the model performance, including sensitivity (Sens), specificity (Spec), Matthews correlation coefficient (MCC) and overall accuracy (OA), as follows:

13 $$ Sens=\frac{TP}{TP+ FN} $$

14 $$ Spec=\frac{TN}{TN+ FP} $$

15 $$ MCC=\frac{TP\times TN- FP\times FN}{\sqrt{\left( TP+ FP\right)\times \left( TP+ FN\right)\times \left( TN+ FP\right)\times \left( TN+ FN\right)}} $$

16 $$ OA=\frac{TP+ TN}{TP+ FN+ FP+ TN} $$

where *TP* represents the numbers of the correctly identified positives, *TN* represents the numbers of correctly identified negatives, *FP* represents the numbers of the negatives identified as positives, *FN* represents the numbers of the positives identified as negatives. In addition, to assess the generalization performance of the model, the receiver operating characteristic (ROC) curves were used. The AUC is the area calculated under ROC curve plotted by FP rate vs TP rate, which is a quantitative indicator of the robustness of the model. Its values range from 0 to 1.

**Fig. 1:**
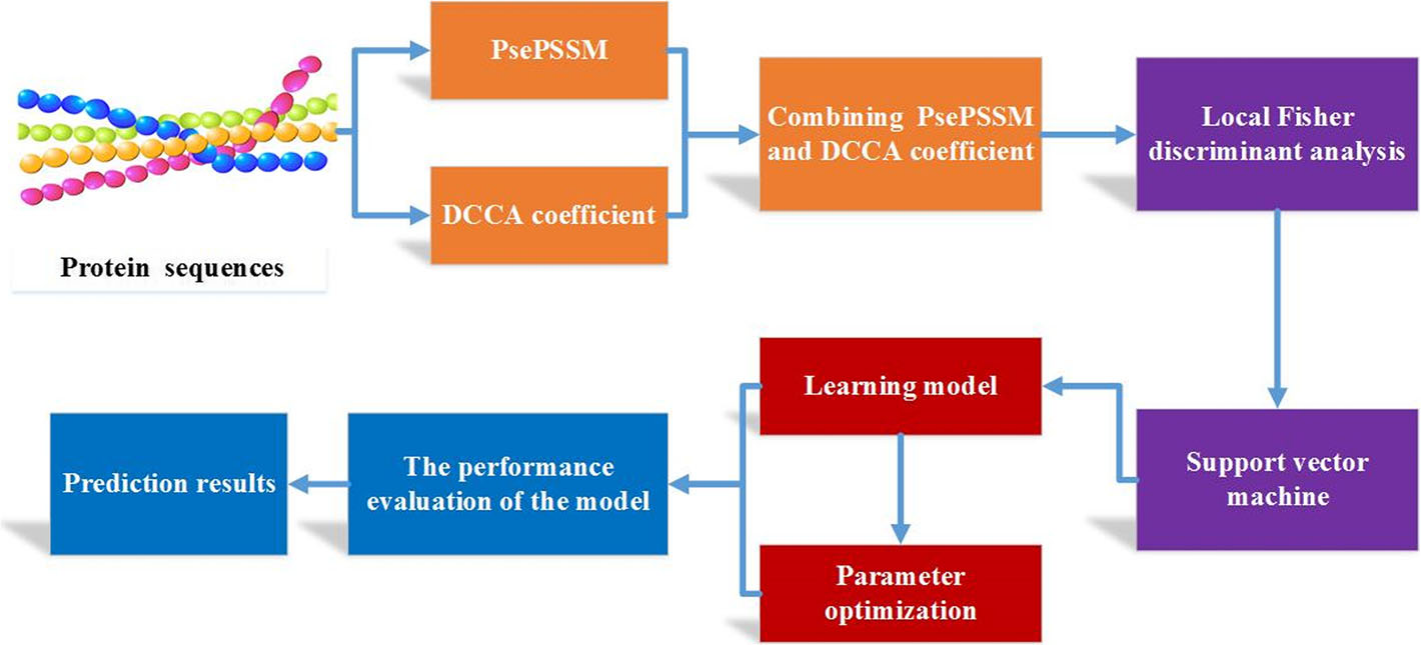
Flowchart of PsePSSM-DCCA-LFDA prediction method

For convenience, the method is called PsePSSM-DCCA-LFDA in this paper, which is used to predict apoptosis protein subcellular localization. To provide an intuitive picture, the flowchart of PsePSSM-DCCA-LFDA method is shown in Fig. 1. We have implemented it in MATLAB R2014a.

The PsePSSM-DCCA-LFDA prediction model is detailed below:

1. Input CL317 dataset and ZW225 dataset, respectively, which contain apoptosis protein sequences and the class label corresponding to all kinds of proteins;
2. The 20 + 20 × *ξ*dimension feature vector is generated by PsePSSM algorithm. Using DCCA coefficient, the protein sequence is extracted to generate 190 dimension feature vectors. By combining these two methods, the two different apoptosis protein datasets generate the corresponding feature extraction matrices of*X* = 317 × (190 + (20 + 20 × *ξ*))and *X* = 225 × (190 + (20 + 20 × *ξ*)), respectively;
3. Using the LFDA method to solve ${T}_{LFDA}=\underset{T\in {R}^{d\times r}}{\arg \max}\kern0.4em \left[ tr\left({\left({T}^T{S}^{(w)}T\right)}^{-1}{T}^T{S}^{(b)}T\right)\right]$, the numerical matrix *X* extracted in 2) is reduced dimension by $Z={T}_{LFDA}^{\hbox{'}}X$, and the matrix *Z* is obtained by removing the redundant information in the apoptosis protein sequences;
4. The matrix *Z* after dimensionality reduction are input into the SVM classifier, and the protein subcellular localization prediction is performed by jackknife test;
5. According to the accuracy of prediction, the optimal parameters of the model are selected, including the *ξ* values and*S*values of parameters, the selection of the dimension reduction algorithm and the dimensionality;
6. Calculate the Sens, Spec, MCC, OA and AUC values of the model, and evaluate prediction performance of the model;
7. Using the independent testing dataset ZD98 to test the PsePSSM-DCCA-LFDA prediction model.

## Results

### Selection of optimal parameter *ξ* and*S*

In this study, the apoptosis protein sequences are extracted by the fusion PsePSSM algorithm and the DCCA coefficient algorithm, and obtain the feature information in the protein sequences. It is worth noting that both the PsePSSM algorithm and the DCCA coefficient algorithm can control the validity of the algorithm to extract the feature information of the protein sequence by adjusting some of the parameters in the algorithm. How to get the best parameters of these two feature extraction algorithms is very important for us to construct a protein subcellular localization prediction model. In order to discover the merits of the feature parameters, we use CL317 and ZW225 datasets as the research object, the best parameters of the model are selected by the prediction accuracy under different parameters. In this paper, the PsePSSM algorithm is used to carry out feature extraction on protein sequences, and the *ξ* value indicates the sequence-order information of the amino acid residues in the protein sequence. If the *ξ* value is set too large, the feature vector dimension of the protein sequence is too high, resulting in more redundant information, which affects the prediction effect. If the *ξ* value is set too small, the feature vector contains very little sequence information, and the features of the protein sequence of the apoptosis protein dataset cannot be extracted comprehensively. To find the optimal *ξ* value in the model, set the *ξ* values from 0 to 10 in turn. For the different *ξ* values, the apoptosis protein datasets CL317 and ZW225 are classified by SVM respectively. The SVM is used to select the radial basis function (RBF) and the results are tested by jackknife method. The overall prediction accuracy of each class protein and overall prediction accuracy in the apoptosis protein datasets are obtained, as are shown in Tables 1 and 2.

**Table 1:** Prediction results of selecting different *ξ* on CL317 by jackknife test

	*ξ*										
Locations	Jackknife test (%)										
	0	1	2	3	4	5	6	7	8	9	10
Cy	88.4	90.2	90.2	92.9	92.0	92.9	92.9	92.9	92.9	92.9	92.9
Me	76.4	83.6	81.8	83.6	83.6	81.8	83.6	83.6	87.3	87.3	87.3
Mi	38.2	55.9	58.8	70.6	64.7	64.7	64.7	64.7	64.7	64.7	67.7
Se	47.1	76.5	76.5	76.5	76.5	76.5	76.5	76.5	76.5	76.5	76.5
Nu	51.9	57.7	63.5	73.1	76.9	73.1	73.1	71.2	71.2	71.2	73.1
En	85.1	85.1	85.1	85.1	85.1	85.1	85.1	85.1	85.1	85.1	85.1
OA	72.2	78.5	79.5	83.6	83.3	82.6	83.0	82.6	83.3	83.3	83.9

**Table 2:** Prediction results of selecting different *ξ* on ZW225 by jackknife test

	*ξ*										
Locations	Jackknife test (%)										
	0	1	2	3	4	5	6	7	8	9	10
Cy	74.3	81.4	80.0	81.4	81.4	80.0	81.4	80.0	80.0	80.0	80.0
Me	93.3	91.0	87.6	88.8	86.5	85.4	85.4	85.4	85.4	85.4	85.4
Mi	16.0	32.0	24.0	40.0	44.0	44.0	44.0	40.0	36.0	36.0	36.0
Nu	48.8	63.4	61.0	68.3	68.3	75.6	75.6	75.6	75.6	75.6	75.6
OA	70.7	76.4	73.3	77.3	76.9	77.3	77.8	76.9	76.4	76.4	76.4

Table 1 shows that the OA of CL317 dataset are different with constant change of *ξ* value. The highest prediction accuracy of mitochondrial proteins reach 70.6% when *ξ* = 3, which is 32.4 and 14.7% higher than when *ξ* values are 0 and 1, respectively. The prediction accuracy of membrane proteins is 87.3% when *ξ* = 10. The OA of CL317 dataset reach 83.6 and 83.9% when *ξ* = 3 and *ξ* = 10, respectively, higher than that when *ξ* values are taking other values.

Table 2 shows that the OA of ZW225 dataset are different with constant change of *ξ* value. For cytoplasmic proteins, the highest predictive accuracy is 81.4% when *ξ* values are 1, 3, 4 and 6, respectively. For membrane proteins, the highest prediction accuracy is 93.3% when *ξ* = 0, which is 7.9% higher than when *ξ* = 5. From the overall prediction accuracy, when *ξ* values are 3 and 6, the OA of ZW225 dataset reach 77.3 and 77.8%, respectively, which is 6.6 and 7.1% higher than that when *ξ* = 0.

**Fig. 2:**
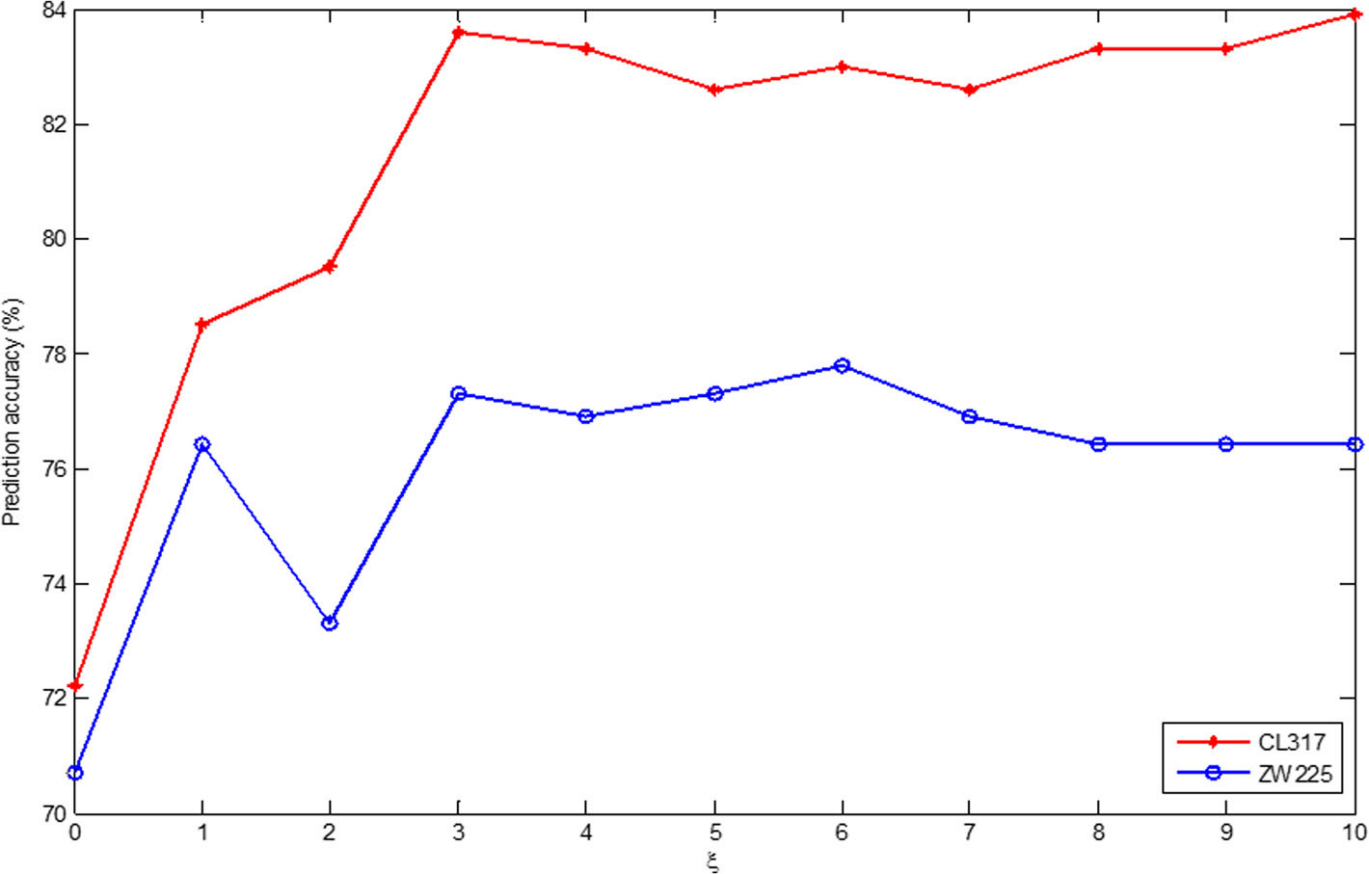
Effect of selecting different values of *ξ* on CL317 and ZW225 datasets by jackknife test

To select the optimal parameters of the PsePSSM algorithm in the subcellular prediction model of apoptosis proteins, CL317 and ZW225 datasets are selected as the training datasets. Fig. 2 shows the OA changes when different *ξ* values are chosen in CL317 and ZW225 datasets. It can be seen from Fig. 2 that the prediction accuracy of the two datasets is changing with the change of the *ξ* value. In addition, CL317 and ZW225 datasets reach the highest accuracy, when*ξ* = 3 and*ξ* = 6, respectively. But in order to unify the model parameters, *ξ* = 3 is chosen in the model. Therefore, the PsePSSM algorithm is used to extract the protein sequence, and each protein sequence to obtain 20 + 20 × *ξ* = 20 + 20 × 3 = 80 dimension feature vector.

In the feature extraction process by using DCCA coefficient, the selection of *S*value has a crucial influence on the construction of the model. *S*is used to determine the length of each overlapping portion of the detrended cross-correlation analysis. Because the length of the shortest protein sequence in the benchmark dataset is 50, the maximum value allowed for *S* is 49. To find the optimal *S*value in the model, set *S*values from 5 to 49 in turn. For the different *S*values, the apoptosis protein datasets CL317 and ZW225 are classified by SVM respectively. The SVM is used to select RBF and the results are tested by jackknife method. The prediction accuracy of each class protein and the overall prediction accuracy in the apoptosis protein datasets are obtained, as are shown in Tables 3 and 4.

**Table 3:** Prediction results of selecting different *S* on CL317 by jackknife test

	*S*									
Locations	Jackknife test (%)									
	5	10	15	20	25	30	35	40	45	49
Cy	96.4	90.2	92.0	90.2	95.5	97.3	97.3	95.5	95.5	96.4
Me	83.0	83.0	83.0	83.0	83.0	83.0	83.0	83.0	83.0	83.0
Mi	80.0	87.3	83.6	87.3	83.6	87.3	89.1	90.9	92.7	92.7
Se	44.1	52.9	82.4	79.4	88.2	85.3	85.3	82.4	82.4	82.4
Nu	46.2	63.5	63.5	65.4	61.5	61.5	63.5	65.4	65.4	67.3
En	64.7	52.9	52.9	52.9	70.6	70.6	76.5	76.5	64.7	58.8
OA	76.0	78.2	81.4	81.4	83.9	84.9	85.8	85.5	85.2	85.5

**Table 4:** Prediction results of selecting different *S* on ZW225 by jackknife test

	*S*									
Locations	Jackknife test (%)									
	5	10	15	20	25	30	35	40	45	49
Cy	85.7	81.4	80.0	85.7	84.3	85.7	84.3	85.7	85.7	87.1
Me	84.3	86.5	86.5	91.0	91.0	89.9	89.9	91.0	89.9	89.9
Mi	28.0	36.0	48.0	52.0	56.0	56.0	56.0	56.0	56.0	56.0
Nu	48.8	68.3	70.7	61.0	58.5	58.5	70.7	75.6	75.6	73.2
OA	72.0	76.0	77.3	79.6	79.1	79.1	80.9	82.7	82.2	82.2

Table 3 shows that the OA of CL317 dataset are different with constant change of *S* value. The accuracy of cytoplasmic proteins reach 97.3% when *S* = 30 and *S* = 35, respectively. The highest prediction accuracy of mitochondrial proteins reach 92.7%, when *S* = 45 and *S* = 49, respectively, which is 12.7% higher than when*S* = 5. The accuracy of nuclear proteins is 67.3% when *S* = 49, which is 21.1% higher than when*S* = 5. The accuracy of secreted proteins is 88.2% when *S* = 25. From overall prediction accuracy, the OA of CL317 dataset is 85.8% when *S* = 35, which is 9.8% higher than when *S* = 5.

Table 4 shows that the OA of ZW225 dataset are different with constant change of *S*value. The accuracy of cytoplasmic proteins reach 87.1% when*S* = 49. The highest prediction accuracy of membrane proteins reach 91.0% when *S* values are 20, 25 and 40, respectively. The accuracy of nuclear proteins reach 75.6% when *S* = 40 and *S* = 45, respectively. From the overall prediction accuracy, the OA of ZW225 dataset is 82.7% when *S* = 40, which is higher than other parameters.

**Fig. 3:**
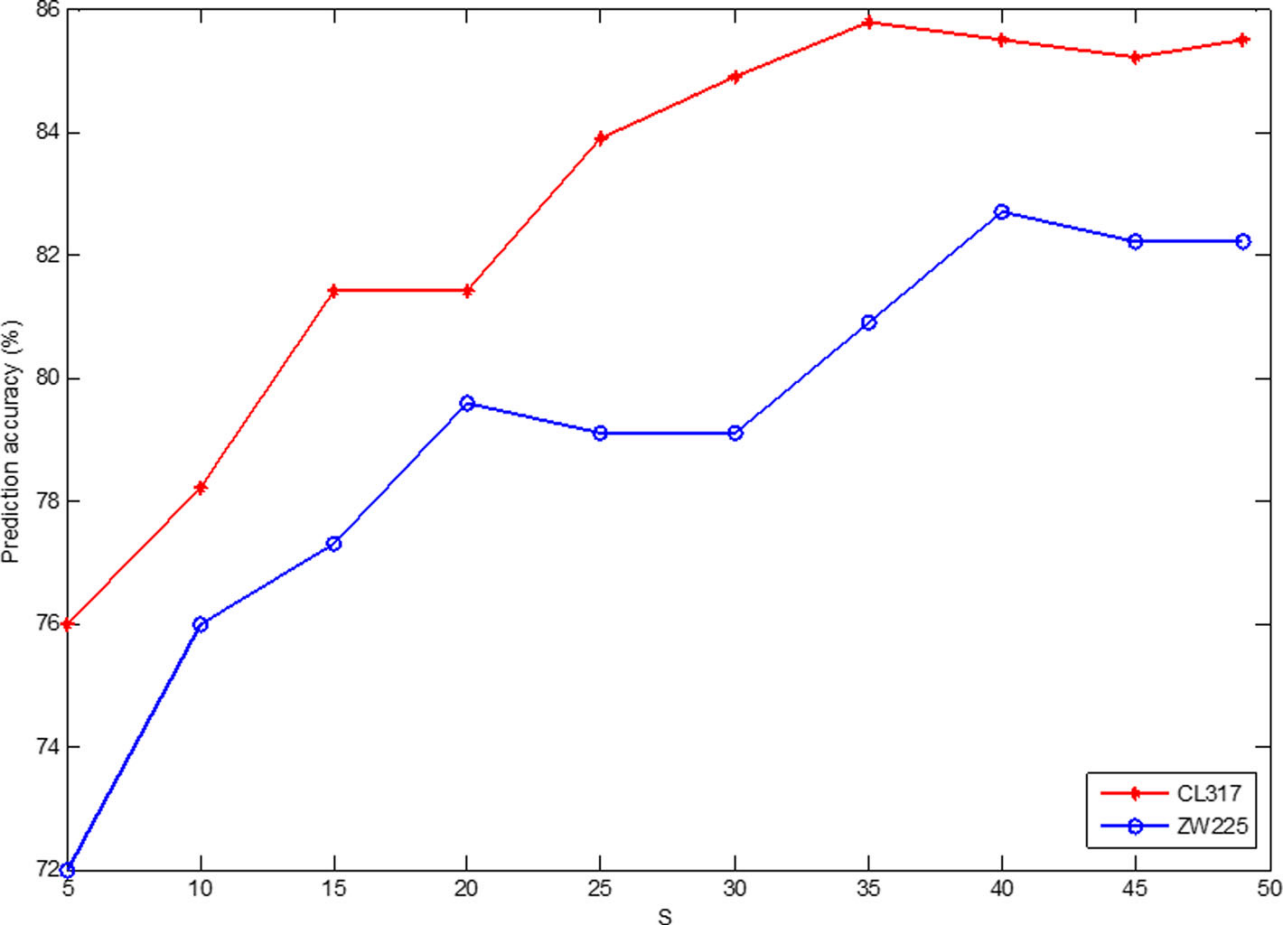
Effect of selecting different values of *S* on CL317 and ZW225 datasets by jackknife test

In our current study, two apoptosis protein datasets CL317 and ZW225 are selected as the training datasets. To determine the optimal parameters of DCCA coefficient algorithm in the model, Fig. 3 shows the change of OA in CL317 dataset and ZW225 dataset by choosing different*S*. It can be seen from Fig. 3 that*S*values are different for the highest overall prediction accuracy of two datasets. The average overall prediction accuracy of CL317 and ZW225 datasets is the highest when *S* = 40. That is, the DCCA coefficient algorithm chooses optimal parameter*S* = 40. At this time, the 190 dimension feature vector can be obtained by extracting each protein sequence by DCCA coefficient method.

### Selection of dimensionality reduction method and optimal dimension

The increasing dimension of the dataset makes the classification more difficult and the development to a certain extent can cause curse of dimensionality. For high-dimensional data, firstly, dimensionality reduction is carried out, and then data after dimensionality reduction is input into the learning system. In order to achieve the ideal protein subcellular localization prediction accuracy, the PsePSSM and DCCA coefficients are first fused to extract features of the protein sequences. In the discussion of section 3.1, in the PsePSSM algorithm, select *ξ* = 3. In the DCCA coefficient algorithm, select *S* = 40. At this time, each protein sequence in the dataset generates a (20 + 20 × 3) + 190 = 80 + 190 = 270 dimension feature vector.

Then, PCA (Principal Component Analysis) [90], Laplacian Eigenmaps [91], AKPCA (Adaptive Kernel Principal Component Analysis) [92] and LFDA (Local Fisher Discriminant Analysis) dimensionality reduction method are used to compare the effect of protein subcellular localization overall prediction accuracy by using these four dimensionality reduction methods. In this study, we use the SVM to classify with the radial basis kernel function, and the results are tested by jackknife method. The overall prediction accuracy of subcellular localization of two apoptosis protein datasets are obtained with different dimensionality reduction methods and under different dimensions, as shown in Tables 5 and 6.

**Table 5:** Prediction results of subcellular localization of the CL317 dataset by selecting different dimensionality reduction methods and different dimensions

	Dimensions									
Algorithms	Jackknife test (%)									
	10	20	30	40	50	60	70	80	90	100
PCA	79.5	84.9	89.0	89.3	87.7	86.1	85.8	83.9	82.6	80.4
Laplacian	63.4	73.8	79.2	82.6	84.9	84.5	82.3	80.8	78.5	74.4
AKPCA	78.2	84.5	87.7	89.0	89.6	88.6	87.4	88.3	86.8	86.4
LFDA	99.7	98.7	98.7	98.4	98.4	97.8	97.5	97.5	97.2	97.2

**Table 6:** Prediction results of subcellular localization of the ZW225 dataset by selecting different dimensionality reduction methods and different dimensions

	Dimensions									
Algorithms	Jackknife test (%)									
	10	20	30	40	50	60	70	80	90	100
PCA	74.2	79.1	85.8	84.9	83.6	81.3	78.7	78.2	77.8	74.7
Laplacian	69.3	80.0	82.2	80.9	82.2	77.8	73.8	72.0	68.0	67.1
AKPCA	74.7	82.2	84.0	86.2	84.9	83.1	80.9	80.9	77.3	78.2
LFDA	99.6	99.6	99.6	99.6	99.6	99.6	99.6	99.6	99.1	99.1

**Fig. 4:**
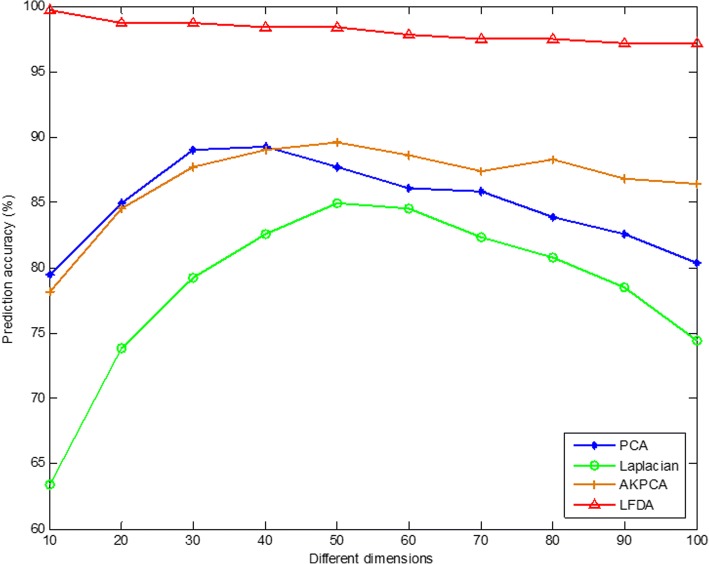
Effects of selecting four different dimensionality reduction methods and different dimensions on the overall prediction results of subcellular localization in CL317 dataset

As can be seen from Table 5, for the CL317 dataset, choosing different dimensionality reduction methods and dimensions have a significant effect on the accuracy of protein subcellular prediction. When Laplacian Eigenmaps and AKPCA method are used to reduce dimension, the dimension is 50, and CL317 dataset obtains the highest overall prediction accuracy, which is 84.9 and 89.6%, respectively. When PCA method is used to reduce dimension and dimensionality chooses 40, the highest overall prediction accuracy of the CL317 dataset is 89.3%. When LFDA method is used to reduce dimension and dimensionality chooses 10, the overall prediction accuracy is the highest, which is 99.7%. It shows that when choosing different dimensionality reduction methods, getting the best dimension for the CL317 dataset is different. By comparing the overall prediction accuracy by different dimensionality reduction methods with different dimensions, we can find that when the LFDA dimensionality reduction method is adopted, the dimensionality is 10, the overall prediction accuracy is the highest, 10.1% higher than when AKPCA dimensionality reduction method is used and the dimension is 50. It can be more intuitively found in Fig. 4 for the CL317 dataset, when the LFDA dimensionality reduction method is selected, the highest overall prediction accuracy of the model is achieved when dimension is 10.

**Fig. 5:**
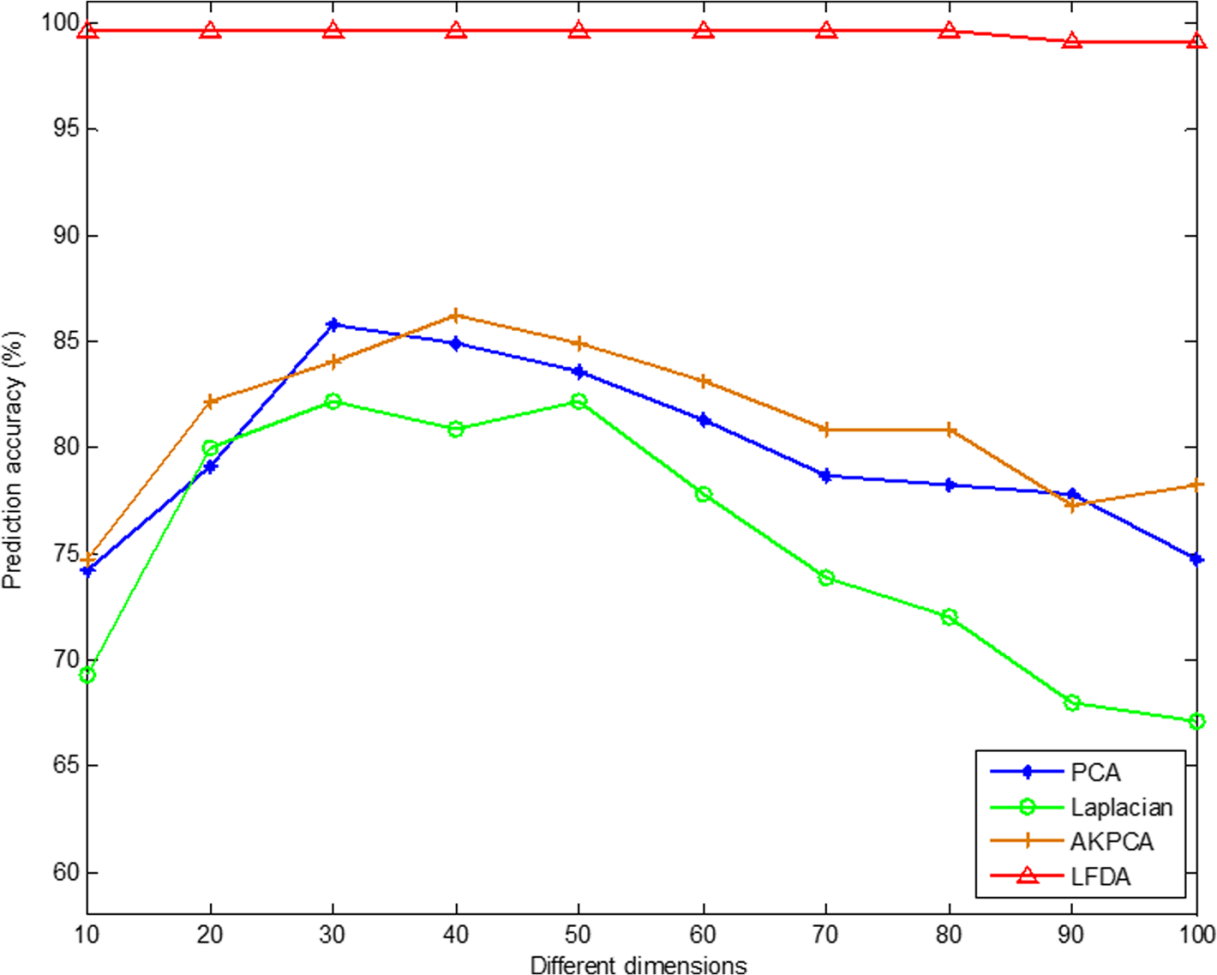
Effects of selecting four different dimensionality reduction methods and different dimensions on the overall prediction results of subcellular localization in ZW225 dataset

As can be seen from Table 6, for the ZW225 dataset, choosing different dimensionality reduction methods and dimensions has a significant effect on the accuracy of protein subcellular prediction. When PCA method is selected to reduce the dimensionality and dimension chooses 30, the highest overall prediction accuracy of 85.8% is achieved in the ZW225 dataset. When using the Laplacian Eigenmaps method to reduce dimension, and dimensionality chooses 30 or 50, the overall prediction accuracy of the dataset is the highest, which is 82.2%. When using the AKPCA method to reduce dimension, and dimensionality chooses 40, the highest overall prediction accuracy is 86.2%. When using the LFDA method to reduce dimension, and dimensionality chooses 10, 20, 30, 40, 50, 60, 70 or 80, the highest overall prediction accuracy is 99.6%. It indicates that the choice of the optimal dimension is closely related to the use of dimensionality reduction methods. In this paper, by comparing the overall prediction accuracy by different dimensionality reduction methods with different dimensions, it can be found that when the LFDA dimensionality reduction method is adopted and the dimension is 10, the overall prediction accuracy is the highest, 17.4% higher than when Laplacian Eigenmaps dimensionality reduction method is used and dimension is 30. It can be more intuitively found in Fig. 5 for the ZW225 dataset, when the LFDA dimensionality reduction method is selected, the highest overall prediction accuracy of the model when dimension is 10, 20, 30, 40, 50, 60, 70 or 80. Since the two apoptosis protein datasets CL317 and ZW225 are selected as the training set, in order to unify the parameters of the model, the LFDA dimensionality reduction method is adopted in this paper, and the optimal dimension is 10-dimensional.

### Effect of feature extraction algorithm on results

**Fig. 6:**
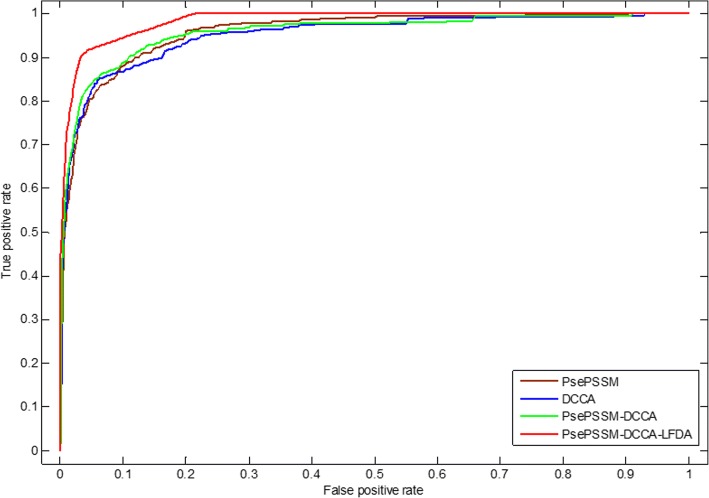
The ROC curves of four different feature extraction methods on dataset CL317

**Fig. 7:**
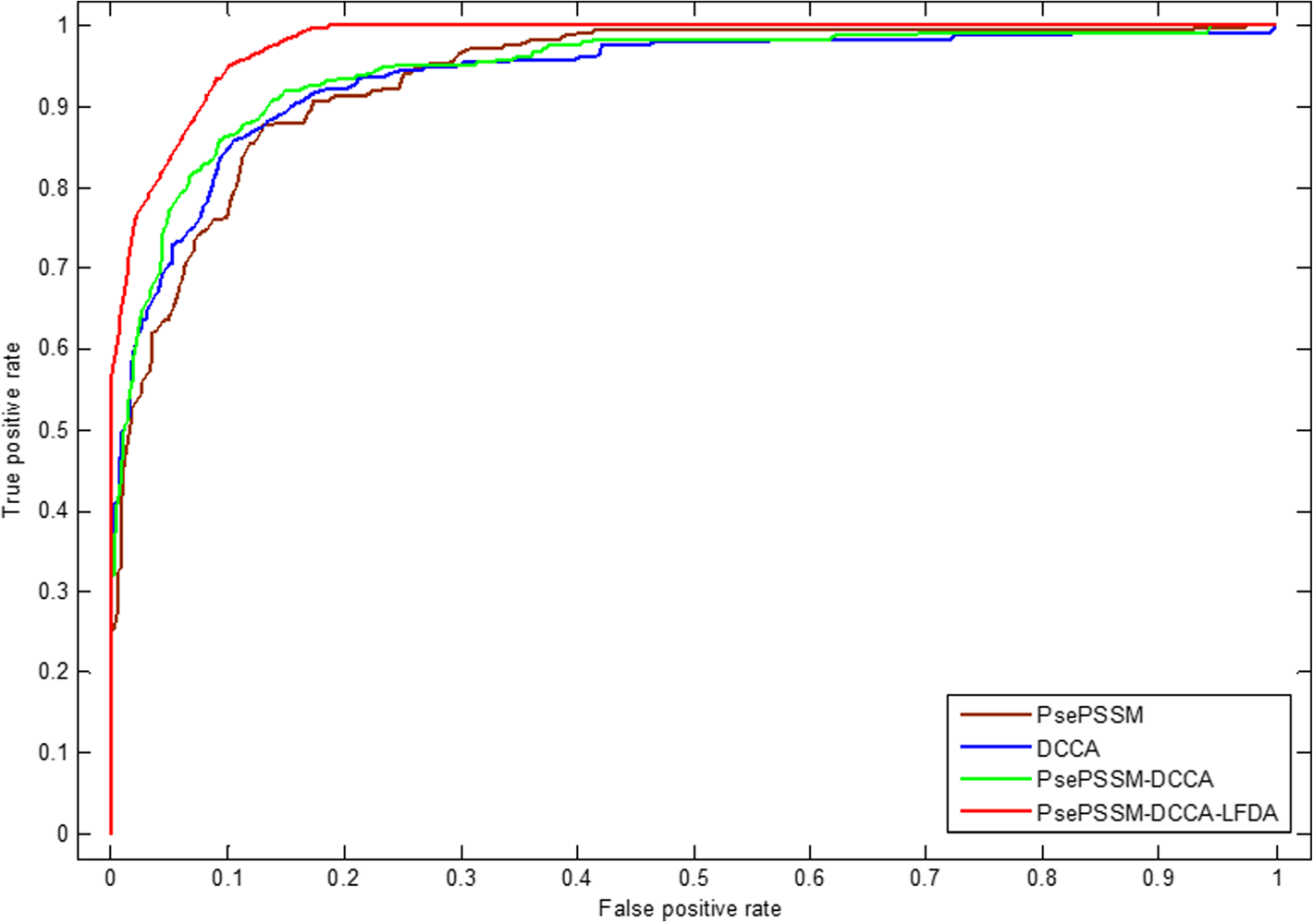
The ROC curves of four different feature extraction methods on dataset ZW225

Feature extraction method converts character representation of a protein sequence into a numerical representation, which uses the corresponding feature vector to represent protein sequence information. PsePSSM method can get homology and sequence information of amino acids in the protein sequences. DCCA coefficient method is an extension of the DCCA and the DFA (detrended fluctuation analysis). Here, only the evolutionary represented in the form of PSSM is adopted as the considered properties. The PsePSSM algorithm and the DCCA coefficient method are combined to obtain more protein sequence information, but this will obtain high-dimensional features to make the model worse, which contain more redundant variables. LDFA dimensionality reduction method use local within-class scatter matrix and local between-class scatter matrix to remove the redundant information based on the feature information of the protein sequences in the dataset and the corresponding class labels. In this paper, the optimal feature extraction algorithm is selected by comparing the influence of different feature extraction methods on the prediction results. Two different predicted results of the two apoptosis protein datasets CL317 and ZW225 are shown in Tables 7 and 8. Furthermore, we analyze the robustness of the model under different feature extraction algorithms, which use ROC curve. As we know, the ROC curve is used in positive vs negative (two classes) classification. But apoptosis proteins subcellular localization prediction is a multi-class prediction problem. We first use the one-versus-rest (OVR) strategy to transform the multi-classification problem into two-classification problems. One of the classes is selected as positive samples i.e. “positive” one and other classes as negative samples [69]. Then for these two-classification true positive rate and false positive rate, the average of them was taken as the final result [43]. Figures 6 and 7 are the ROC curves obtained by four different feature extraction methods for the CL317 dataset and ZW225 dataset, respectively.

**Table 7:** Prediction results of different feature extraction methods on CL317 by jackknife test

	Locations						
Algorithms	Jackknife test (%)						
	Cy	Me	Mi	Se	Nu	En	OA
PsePSSM	92.9	85.1	83.6	70.6	73.1	76.5	83.6
DCCA	95.5	83.0	90.9	82.4	65.4	76.5	85.5
PsePSSM-DCCA	93.8	85.1	90.9	82.4	76.9	70.6	86.8
PsePSSM-DCCA-LFDA	99.1	100	100	100	100	100	99.7

**Table 8:** Prediction results of different feature extraction methods on ZW225 by jackknife test

	Locations				
Algorithms	Jackknife test (%)				
	Cy	Me	Mi	Nu	OA
PsePSSM	81.4	88.8	40.0	68.3	77.3
DCCA	85.7	91.0	56.0	75.6	82.7
PsePSSM-DCCA	88.6	88.8	56.0	87.8	84.9
PsePSSM-DCCA-LFDA	100	98.9	100	100	99.6

Table 7 shows that the OA of CL317 dataset are different, which use different feature extraction algorithms. The OA of PsePSSM algorithm reach 83.6%, which is 1.9% lower than DCCA coefficient algorithm. The OA of PsePSSM-DCCA algorithm is 86.8%, which is 3.2, 1.3% higher than PsePSSM and DCCA coefficient algorithm, respectively. The LFDA algorithm is used to reduce the dimensionality after two algorithms. The accuracy of each class has been obviously improved by using LFDA algorithm and OA of CL317 dataset reach 99.7%. For PsePSSM algorithm, the accuracy of secreted proteins reach 70.6%, which is lower than DCCA coefficient, PsePSSM-DCCA and PsePSSM-DCCA-LFDA algorithm, respectively. The accuracy of secreted proteins is 100% by PsePSSM-DCCA-LFDA algorithm, which is 29.4% higher than the PsePSSM method. Fig. 6 shows that PsePSSM-DCCA-LFDA reach largest coverage area of the ROC curve, whose AUC value is 0.9842. In addition, the AUC values of PsePSSM, DCCA coefficient and PsePSSM-DCCA are 0.9591, 0.9520 and 0.9587, respectively.

Table 8 shows that the OA, accuracy of each class are different for ZW225 dataset with different feature extraction algorithms. The OA of PsePSSM algorithm reach 77.3%, which is 5.4, 7.6 and 22.3% lower than DCCA coefficient algorithm, PsePSSM-DCCA algorithm and PsePSSM-DCCA-LFDA algorithm, respectively. The OA of PsePSSM-DCCA algorithm is 84.9%, which is 7.6, 2.2% higher than PsePSSM and DCCA coefficient algorithm, respectively. Using the LFDA algorithm to reduce the dimensionality, the PsePSSM-DCCA algorithm as feature extraction method, the prediction accuracy of the four kinds of proteins in the ZW225 dataset has been improved remarkably, and the OA of the model has reach 99.6%. Fig. 7 shows that PsePSSM-DCCA-LFDA reach largest coverage area of the ROC curve, whose AUC value is 0.9805. In addition, the AUC values of PsePSSM, DCCA coefficient and PsePSSM-DCCA are 0.9380, 0.9386 and 0.9464, respectively. Analyzing and comparing the prediction results and robustness of prediction model on CL317 and ZW225 datasets by using four different feature extraction methods, we choose PsePSSM-DCCA-LFDA as feature extraction method in this paper.

### Performance of prediction model

In PsePSSM-DCCA-LFDA prediction model, protein sequence information is extracted by fusing the PsePSSM and DCCA coefficient methods, and then the subcellular localization of apoptosis protein datasets is predicted by SVM based on LFDA dimensionality reduction method. According to the above analysis, when using PsePSSM, *ξ* = 3 is selected, when using DCCA coefficient, *S* = 40is selected. Using the LFDA method to reduce the dimension of the dataset, the optimal dimension chooses 10. The RBF is selected as the kernel function of SVM. In this paper, the most rigorous jackknife test methods are used to test the datasets CL317 and ZW225, the main results are shown in Table 9.

**Table 9:** Prediction performance of datasets CL317 and ZW225 protein subcellular localization on the jackknife test method

	Jackknife test					
Locations	CL317			ZW225		
	Sens (%)	Spec (%)	MCC	Sens (%)	Spec (%)	MCC
Cy	99.1	100	0.99	100	99.4	0.99
Me	100	100	1	98.9	100	0.99
Mi	100	99.6	0.99	100	100	1
Se	100	100	1	–	–	–
Nu	100	100	1	100	100	1
En	100	100	1	–	–	–
OA (%)	99.7			99.6		

As can be seen from Table 9, the OA of CL317 dataset is 99.7% by using jackknife test. The sensitivity of each class is 100% except cytoplasmic proteins. The sensitivity of cytoplasmic proteins is 99.1%. The specificity of each class is 100% except mitochondrial proteins. The OA of ZW225 dataset is 99.6% by using jackknife test. The sensitivity, specificity and MCC of mitochondrial and nuclear proteins are 100, 100% and 1, respectively. The sensitivity of cytoplasmic proteins is 100%, the specificity and MCC are 99.4% and 0.99, respectively.

### Comparison with other methods

In this section, to demonstrate the effectiveness of the proposed method PsePSSM-DCCA-LFDA, we compared with other recently reported prediction methods on the same apoptosis proteins datasets. All the methods are performed using jackknife cross-validation test. Tables 10 and 11 details the comparison of the proposed method and other prediction methods on the CL317 and ZW225 datasets, respectively.

**Table 10:** Prediction results of different methods on CL317 dataset by jackknife test

	Jackknife test (%)						
Methods	Sensitivity for each class (%)						OA (%)
	Cy	Me	Mi	Se	Nu	En	
ID [93]	81.3	81.8	85.3	88.2	82.7	83.0	82.7
ID_SVM [39]	91.1	89.1	79.4	58.8	73.1	87.2	84.2
DF_SVM [21]	92.9	85.5	76.5	76.5	93.6	86.5	88.0
FKNN [40]	93.8	92.7	82.4	76.5	90.4	93.6	90.9
PseAAC_SVM [95]	93.8	90.9	85.3	76.5	90.4	95.7	91.1
EN_FKNN [96]	98.2	83.6	79.4	82.4	90.4	97.9	91.5
DWT_SVM [41]	100	98.2	82.4	94.1	100	100	97.5
Auto_Cova [42]	86.4	90.7	93.8	85.7	92.1	93.8	90.0
APSLAP [97]	99.1	89.1	85.3	88.2	84.3	95.8	92.4
Liu et al. [43]	98.2	96.4	94.1	82.4	96.2	95.7	95.9
PSSM_AC [98]	93.8	90.9	91.2	82.4	86.5	95.7	91.5
DCCA coefficient [46]	91.1	92.7	82.4	76.5	80.8	93.6	88.3
PsePSSM-DCCA-LFDA	99.1	100	100	100	100	100	99.7

**Table 11:** Prediction results of different methods on ZW225 dataset by jackknife test

	Jackknife test (%)				
Methods	Sensitivity for each class (%)				OA (%)
	Cy	Me	Mi	Nu	
EBGW_SVM [15]	90.0	93.3	60.0	63.4	83.1
ID_SVM [39]	92.9	91.0	68.0	73.2	85.8
DF_SVM [21]	87.1	92.1	64.0	73.2	84.0
FKNN [40]	84.3	93.3	72	85.5	85.8
EN_FKNN [96]	94.3	94.4	60.0	80.5	88.0
DWT_SVM [41]	87.1	93.2	64	90.2	87.6
Liu et al. [43]	97.1	98.9	96.0	97.6	97.8
PSSM_AC [98]	82.9	92.1	68.0	78.0	84.0
Auto_Cova [42]	81.3	93.3	85.7	84.6	87.1
PsePSSM-DCCA-LFDA	100	98.9	100	100	99.6

As can be seen from Table 10, the OA of CL317 dataset is 99.7% by using PsePSSM-DCCA-LFDA, which is 2.2–17% higher than other prediction methods. We can find that the overall accuracy by our method is higher than that of ID [93], ID_SVM [39], DF_SVM [21], FKNN [40] and so on. The value of sensitivity for each protein class is listed. For example, the sensitivity of mitochondrial proteins, nuclear proteins, secreted proteins, endoplasmic proteins and membrane proteins eached 100% by our method, while the ID [93] are 85.3, 82.7, 88.2, 83.0 and 81.8%, respectively. For the cytoplasmic proteins, the sensitivity of our method is 99.1%, which is also the highest, which is 12.7% higher than that of the Auto_Cova [42] method. For the CL317 dataset, our proposed method has achieved satisfactory prediction results.

As can be seen from Table 11, the OA of ZW225 dataset is 99.6% using PsePSSM-DCCA-LFDA, which is almost 16.5, 15.6, 13.8 and 12.5% higher than EBGW_SVM [15], DF_SVM [21], FKNN [40], Auto_Cova [42], respectively. Especially for the most difficult case-mitochondrial proteins, the predictive accuracy has improved to 100% by our method, which is 40% higher than that of the EBGW_SVM [15], 36% higher than the prediction accuracy of DF_SVM [21]. It indicates that the model of this paper has excellent properties for the prediction of mitochondrial proteins in apoptosis proteins. In general, for the ZW225 dataset, our proposed method has achieved satisfactory prediction results.

In order to further validate the actual prediction ability of the model, we use the independent testing dataset ZD98 to test the model. When using PsePSSM, *ξ* = 3 is selected. When using DCCA coefficient, *S* = 40 is selected. Using LFDA method to reduce the dimension of the dataset, the optimal dimension chooses 10. The RBF is selected as the kernel function of SVM. The results of the ZD98 dataset are tested by the jackknife cross-validation method and compared with other reported prediction methods. Table 12 shows the predictive results of the subcellular localization on the ZD98 dataset.

**Table 12:** Prediction results of different methods on the independent testing dataset ZD98 by jackknife test

	Jackknife test (%)				
Methods	Sensitivity for each class (%)				OA (%)
	Cy	Me	Mi	Other	
ID [93]	90.7	90.0	92.3	91.7	90.8
ID_SVM [39]	95.3	93.3	84.6	58.3	88.8
DF_SVM [21]	97.7	96.7	92.3	75.0	93.9
FKNN [40]	95.3	96.7	100	91.7	95.9
PseAAC_SVM [95]	95.3	93.3	92.3	83.3	92.9
DWT_SVM [41]	95.4	93.3	53.9	91.7	88.8
APSLAP [97]	95.3	90.0	100	91.7	94.9
Liu et al. [43]	95.3	100	100	91.7	96.9
PSSM_AC [98]	97.7	96.7	100	83.3	95.9
EBGW_SVM [15]	97.7	90.0	92.3	83.3	92.9
DCCA coefficient [46]	93.0	86.7	92.3	75.0	88.9
PsePSSM-DCCA-LFDA	100	100	100	100	100

As can be seen from Table 12, the OA of ZD98 dataset is 3.1–11.2% higher than other methods by using PsePSSM-DCCA-LFDA, which is 9.2% higher than ID [93], 11.2% higher than ID_SVM [39] and DWT_SVM [41]. In addition, the sensitivity of mitochondrial proteins, cytoplasmic proteins and membrane proteins reached 100% by our method, while the ID_SVM [39] are 84.6, 95.3 and 93.3%, respectively. Especially for mitochondrial proteins, the prediction accuracy of DWT_SVM [41] is 53.9%, which is 46.1% lower than that of our method. It shows that our method has achieved good results of the mitochondrial proteins prediction. For the accuracy of the other proteins by the algorithm proposed in this paper is 100%, which is 41.7% higher than the ID_SVM method [39]. In conclusion, the above results indicate that the prediction model we construct can significantly improve the prediction accuracy of protein subcellular localization and has achieved satisfactory prediction results.

## Discussion

In this paper, we propose a novel method for predicting apoptosis protein subcellular localization, called PsePSSM-DCCA-LFDA. When using PsePSSM, *ξ* = 3 is selected. When using DCCA coefficient, *S* = 40 is selected. Using LFDA method to reduce the dimension of the dataset, the optimal dimension chooses 10. The RBF is selected as the kernel function of SVM. The overall prediction accuracy are 99.7, 99.6 and 100% for CL317 dataset, ZW225 dataset and ZD98 dataset by the most rigorous jackknife test, respectively, which is better than other state-of-the-art methods. The OA of CL317 dataset is 99.7% by using PsePSSM-DCCA-LFDA, which is 2.2–17% higher than other prediction methods. The OA of ZW225 dataset is 99.6% by using PsePSSM-DCCA-LFDA, which is 1.8–16.5% higher than other prediction methods. The OA of ZD98 dataset is 3.1–11.2% higher than other methods by using PsePSSM-DCCA-LFDA.

PsePSSM-DCCA-LFDA demonstrated good performance on predicting apoptosis protein subcellular localization, which is better than the state-of-the-art methods. It is mainly due to the following reasons:

1. Both the PsePSSM algorithm and the DCCA coefficient method extract feature information from the PSSM corresponding to the protein sequences. Although both algorithms are data mining the evolutionary information of protein sequences in order to obtain the best numerical representation of the protein sequences, the two algorithms are different. PsePSSM feature extraction takes into account the sequence-order information of the protein sequence. The DCCA coefficient uses the columns in the PSSM as the least squares fitting and the trend elimination as the non-stationary time series to remove the PSSM between the cross-correlation.
2. LFDA can effectively remove redundant information in the protein sequences without losing important information in the apoptosis protein sequence.
3. SVM classification algorithm can deal with high-dimensional data, avoiding over-fitting and effectively removing non-support vector.

Protein subcellular localization information can explain the disease mechanism, provide theoretical basis and solution. Medical studies have found that abnormal subcellular localization of proteins occurs, when cells is cytopathic. Further, abnormally localized proteins provide molecular markers for the early diagnosis of diseases and can become molecular targets for the design of new drugs, which achieve the goal of curing diseases.

Currently our method is still trained on small dataset, because CL317, ZW225 and ZD98 datasets are widely used benchmark datasets, it is difficult to collect large-scale experimentally verified. In the next step, we will build a large-scale protein subcellular dataset for prediction research.

## Conclusion

With the advent of the big data age, the gap between the number of proteins in the public database and its functional annotations is widening. The critical challenge of bioinformatics is to develop automated methods for fast and accurately determining the structures and functions of proteins [94]. In this paper, a novel method for protein subcellular localization prediction is proposed. We use the LFDA dimensionality reduction method and the SVM algorithm to predict the apoptotic protein subcellular localization. Firstly, we fuse the PsePSSM and DCCA coefficient methods to carry out feature extraction on protein sequences. Then, the extracted feature vectors are used to reduce the dimension using LFDA method, and the subcellular localization of apoptosis proteins are predicted by SVM algorithm. By jackknife test, the OA of the three benchmark datasets reach 99.7, 99.6 and 100%, respectively. The results show that the PsePSSM-DCCA-LFDA method has good performance by comparing with others, which use the same benchmark datasets. Since user-friendly and publicly accessible web-server is one of the important factors in building a practical predictive system [78, 88], in order for the convenience of the researchers, we will develop a web-server or standalone version for the prediction method presented in this paper.

## Abbreviations

AKPCA: Adaptive Kernel Principal Component Analysis
CFS: Correlation-based feature selection
Cy: Cytoplasmic proteins
DAGSVM: Direct acyclic graph SVM
DCCA coefficient: Detrended cross-correlation analysis coefficient
DFA: Detrended fluctuation analysis
En: Endoplasmic reticulum proteins
FN: The number of false negatives
FP: The number of false positives
GA: Genetic algorithm
LFDA: Local Fisher discriminant analysis
MCC: Matthews correlation coefficient
Me: Membrane proteins
Mi: Mitochondrial proteins
mLASSO: Multi-label least absolute shrinkage and selection operator
Nu: Nuclear proteins
OA: Overall accuracy
OVO: One-versus-one
OVR: One-versus-rest
PCA: Principal Component Analysis
PsePSSM: Pseudo-position specific scoring matrix
PsePSSM-DCCA-LFDA: Incorporating PsePSSM and DCCA coefficient based on LFDA dimensionality reduction
PSO: Particle swarm optimization
ROC: Receiver operating characteristic
Se: Secreted proteins
Sens: Sensitivity
Spec: Specificity
SVD: Singular value decomposition
SVM: Support vector machine
TN: The number of true negatives
TP: The number of true positives
